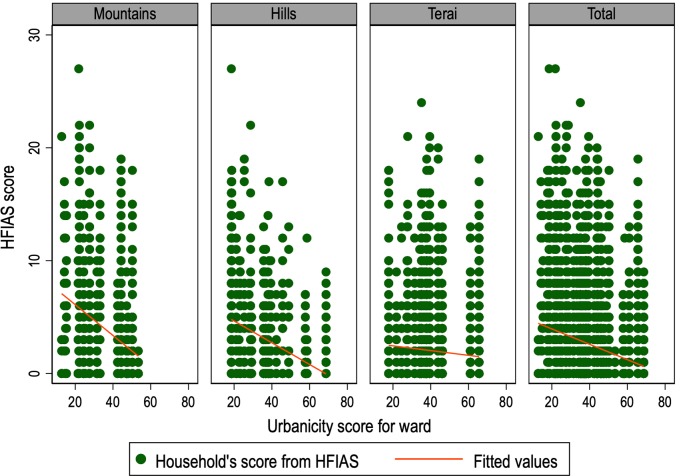# Measuring Community Urbanicity and Its Influence on Household Food Security Across Nepal’s Agroecological Zones

**DOI:** 10.1016/j.cdnut.2024.103773

**Published:** 2024-05-14

**Authors:** Elizabeth Graham, Andrew L Thorne-Lyman, John McGready, Yeeli Mui, Swetha Manohar, Sumanta Neupane, Jessica Fanzo, Keith P West

**Affiliations:** 1 Global Alliance for Improved Nutrition, Washington DC, United States; 2 Department of International Health, Center for Human Nutrition, Johns Hopkins Bloomberg School of Public Health, Baltimore, MD, United States; 3 Department of Biostatistics, Johns Hopkins Bloomberg School of Public Health, Baltimore, MD, United States; 4 Nutrition, Diets and Health Division, International Food Policy Research Institute, Washington DC, United States; 5 Nutrition, Diets and Health Division, International Food Policy Research Institute, New Delhi, India; 6 Climate School, Columbia University, New York, NY, United States

**Keywords:** food security, scale development, urbanicity, Nepal, agricultural livelihood, urbanization

## Abstract

**Background:**

Urbanization influences food systems and food security, but research on these associations in low- and middle-income countries remain limited, partly because of the binary and unstandardized “urban compared with rural” classifications.

**Objectives:**

To develop a community urbanicity scale, to assess its associations with household food security, and to explore whether agricultural occupation modifies this relationship across the 3 agroecological zones (mountain, hill, *Tarai*) of Nepal.

**Methods:**

Data came from a nationally and agroecologically representative, multistaged 2013 agri-food system survey of 4285 households with children <5 y in 63 communities (wards) in Nepal. A novel community-level urbanicity scale was constructed using factor analysis that included 8 domains. Multilevel mixed effects logistic regression was used to assess associations between urbanicity and household food security (measured using the validated Household Food Insecurity Access Scale), and to investigate modifying effects of agricultural occupation.

**Results:**

Urbanicity scores ranged between 13 and 69, of a possible 80 points. Most agricultural households in the mountains (67%) and hills (54%) were categorized food insecure. Increases in urbanicity were negatively associated with food insecurity, controlling for other factors (odds ratio [OR] per 10-unit urbanicity difference OR: 0.82; confidence interval [CI]: 0.71, 0.94; *P* ≤ 0.05). Agricultural occupation may have positively influenced this association though was not a statistically significant effect measure modifier (*P* = 0.07).

**Conclusions:**

The novel scale shows more nuance within Nepal’s agroecological zones, which had similar urbanicity-to-food security relationships as well as overlapping urbanicity score distributions. Research and policy efforts should consider using scales providing more precise urbanicity measurement, and thus informative assessments on its role in predicting food insecurity, especially in agriculturally reliant populations.

## Introduction

As populations worldwide have increased in number and density, a majority of the global population now resides in urban areas [1]. Urbanization is a complex and dynamic process changing the spatial distribution of the world’s populations, and, in effect, changing features (understood to be urban) such as occupations, built infrastructure, market access, and communication [1,2]. It follows that urbanicity is the presence of these features or conditions, defined as the impact of living in an urban area at a given time [3]. Urban residence has generally been linked to positive economic growth, poverty reduction, and human development, but rapid urbanization, particularly in lower income settings, has led to both helpful and harmful effects on human health [4,5].

Urbanicity and food security are inextricably linked, yet the pathways of achieving food security in rapidly urbanizing contexts are not well understood [6]. Food security historically was considered synonymous with agricultural output and production [7], but now a fuller conceptualization spanning physical, social, and economic access, as well as food safety and nutritional adequacy is recognized [8,9], which interplay with facets of urbanization. Food insecurity has been associated with poorer physical health and subjective well-being [10,11], yet remains complexly related to reliance on agricultural livelihoods globally [12], with agricultural livelihoods sometimes protective against and other times a risk factor for food insecurity.

In Nepal, almost one third of national gross domestic product comes from the agricultural sector [13], a main occupation for 33% of men and 70% of women as of 2016 [14]. Just over half (52%) of Nepalese households were categorized as food insecure in 2016, with a higher proportion of rural households (61%) experiencing any food insecurity (mild, moderate, or severe) compared with their urban counterparts [14]. The country still faces relatively high food insecurity prevalence and higher proportions of food insecurity in rural places [15], while also reflecting global risk factors including low education and household income [16]. In Nepal’s hill zone, agricultural work has been associated with improved food security, particularly when those agricultural households grew vegetables, but agricultural households with livestock ownership were associated with lower food insecurity [17]. As of 2018, Nepal was 1 of the least urbanized countries globally [1]; ∼79% of the national population resided in rural areas as of 2020 [18]. Nepal’s Demographic and Health Survey defines “urban” as whether respondents lived within municipality boundaries that were predefined as urban places according to administrative designations [1,14]. In part because of this rural baseline, Nepal is projected to have 1 of the fastest urbanization rates (2.0%/y) between 2018 and 2050 globally [1].

The often used categories of “urban” compared with “rural” fail to reflect the reality that many people now live in settings that are a mix of both urban and rural features [3,19,20]. To advance the measurement of urbanicity, researchers have developed multidimensional scales comprised of multiple variables such as built infrastructure and population density to better explain outcomes along the gradient of urbanicity [19,21]. These scales have differed in their composition as they often draw on existing data collected for other purposes, such as national census and household surveys [22,23]. To date, few studies have explored links between urbanicity scales and food-related outcomes, and none have been applied to nationally representative samples nor in the context of Nepal.

In this article, we sought to: *1*) Develop and apply a validated community-level urbanicity scale across Nepal’s agroecological zones; *2*) Describe the relationship between community urbanicity and household food insecurity at the national level, and whether the relationship varies by agroecological zones; and *3*) Explore whether the relationship between community urbanicity and household food insecurity significantly changes depending on whether households are engaged in agriculture.

## Methods

### Setting and data collection

Data for this article primarily came from the 2013 Policy and Science for Health, Agriculture and Nutrition (PoSHAN) community studies in Nepal, a multiyear nationally and agroecologically representative study. A secondary data source, Nepal’s 2011 National Population and Housing Census [24], contributed 1 variable (population density). The first year of the PoSHAN study (2013) was used as it was the closest year to the 2011 Nepal Census.

The PoSHAN study was conducted to assess community, household, and individual factors that help describe the relationship between agricultural food production, extension service, food security, dietary, health and nutritional status in households with preschool-aged children across the country’s agroecological zones, as well as the influence of policy and program intervention on nutrition outcomes. The study was designed to capture the distinct variation in population density, livelihood, and topography across the 3 agroecological zones (mountains, hills, *Tarai*) of Nepal. The mountain zone includes some of the highest mountain peaks in the world and is characterized by severe terrain that hinders most ground transportation [14]. The hill zone includes the capital city, Kathmandu, and other urban areas that have rapidly developed in previous decades. The *Tarai,* or plains, is where the vast majority of the country’s agricultural production occurs [13]. Previous findings from the PoSHAN study report associations between agriculture, food security, seasonality, and socioeconomic status to diet and nutritional status of children and mothers across and within specific agroecological zones [[25], [26], [27], [28], [29], [30], [31]].

Briefly, a sampling frame was constructed by contiguously listing all 75 districts from west to east within each of the 3 agroecological zones. Within districts, respective village development committees (VDCs, subdistricts) were listed alphabetically, and 7 VDCs were systematically sampled following a random start from each zonal list, yielding a total of 21 VDCs. Within each of the selected VDCs, 3 of 9 wards listed by population size were systematically selected following a random start. This design led to a population-weighted, representative sample of 21 communities (wards) within each zone, thus 63 communities overall. This sampling procedure was designed with a focus on households in more rural and agriculturally reliant spaces, thus excluded all 58 municipalities (cities). In 2013 and subsequent survey years, all households in sampled communities were visited and those with preschool-aged children (≤60 mo) and recently married couples within the past 2 y were enrolled for study [32].

Data from different levels were used for this analysis, including ward enumeration rosters, household observations, and interviews with heads of household. Household surveys were conducted between May and September of 2013, when a total of 4379 households were found to be eligible, of which 4286 households consented and completed interviews. A total of 4285 households (residing across the 63 communities) were included in this analysis.

Ethical clearance for this article as well as the larger PoSHAN study was given by the Institutional Review Board at the Johns Hopkins University Bloomberg School of Public Health. Ethical approval for original data collection was also obtained from the Nepal Health Research Council, with ethical conduct of research and consent procedures training with field staff described elsewhere [32].

### Indicators used

Food insecurity, the main outcome of interest, was measured using an indicator developed from the Household Food Insecurity Access Scale (HFIAS) indicator. The HFIAS is a cross-culturally validated experiential scale which reflects the “access” dimension of food security, and asks about behaviors and psychological manifestations related to insecure access to food in the last 30 d [33,34]. Possible scores range between 0 and 27, which can inform a categorical variable where the prevalence of food insecurity is reported through 4 ordered categories: food secure, mildly food insecure, moderately food insecure, and severely food insecure. For the current regression analysis, categories were collapsed to 1 “food insecure” category compared with “food secure”, the latter including scores of 1 (an affirmative, “rarely” response to the first HFIAS question) and 0.

Agricultural occupation was explored as a possible effect measure modifier using a household survey question that asked the head of household to report their main occupation. Original survey question grouped responses into 9 possible categories: Not working; Retired; Student; Non-earning occupation (housewife/female community health volunteer); Wage employment; Business/trader/self-employment; Salaried worker; Agriculture/livestock/poultry/aquaculture; Other. On the basis of these categories, a binary variable was formed for the current analysis, with “agriculture/livestock/poultry/aquaculture” compared with all other occupation categories.

Community urbanicity, the main exposure of interest, was measured using a novel urbanicity scale and applied to each of the 63 communities. A total of 23 variables were initially considered for the scale based on exploratory analyses of available data and a review of urbanicity and scale development literature; PoSHAN’s 2013 panel survey was used to generate 22 variables, and the 2011 Nepal Population and Housing Census was used to generate 1 district level variable (population density), all listed in Supplemental Table 1. Variables were assembled within 8 domains, based largely on a scale developed and validated for use in India, Ethiopia, and Peru [35]: Population density, economic activity, built environment, markets, communication, education, diversity, and health services. Five of these variables represented similar information: 3 variables captured agricultural employment in slightly different ways (Supplemental Table 1, 2a–2c), and 2 variables captured mobile phone ownership for different family members (Supplemental Table 1, 10a-10b); 6 scale iterations were created by interchanging these 5 variables. Mixed effects analysis of variance (ANOVA) was used to compare the 6 scale iterations, showing ∼99% of the total variance was found within scales rather than between them (intraclass correlation [ICC]: 0.99; SE: 0.002). This shows empirically that these different scale iterations were measuring the same construct—economic activity and communication—and that all performed similarly. Scale 3 was chosen as it comprised commonly collected and publicly available variables, and was similar to other validated urbanicity scales in the literature [21,35,36].

Both the domain correlation matrix (Supplemental Figure 1) and Cronbach’s alpha were employed to assess the scale’s performance and guide which variables were removed, because of unnecessary or redundant items, and to assess reliability. Using a stepwise removal of these, 14 variables grouped within 8 domains remained in the final scale, shown in Table 1. The final scale had a Cronbach alpha of 0.82, and an average interitem correlation of 0.24. Item-test correlations of the 8 domains showed good internal consistency (all values >0.40) across all domains. Principal Component Analysis (PCA) was then used to reduce the dimensionality. Supplemental Figure 2 illustrates eigenvalues after PCA.

**TABLE 1:** Complete scale algorithm. Scoring system for calculating community’s urbanicity

Domain	Variable	Scoring scheme	Points possible
Population density	Population density at district level (Nepal’s 2011 Census)	1–50	1
		51–100	2
		101–150	3
		151–200	4
		201–300	5
		301–400	6
		401–500	7
		501–600	8
		601–700	9
		701+	10
Economic activity	Percent of households with one or more adults reporting agricultural occupation^1^	10 points – 10 ∗ % of households with 1 or more adults reporting agricultural occupation	0–10
Built environment	Presence of paved roads	Any paved roads in ward	0 or 1
	Presence of bus stops	Any bus stop in ward	0 or 1
	Percent of households with flush toilet	4 points ∗ % of households with flush toilet	0–4
	Percent of households with electricity	4 points ∗ % of households with electricity	0–4
Markets	Presence of permanent bazaar	5 points ∗ any permanent bazaar market in ward	0 or 5
	Presence of Haat bazaar	5 points ∗ any Haat bazaar in ward	0 or 5
Communication	Percent of households owning ≥1 TVs	5 points ∗ % of households owning ≥1 TVs	0–5
	Percent of households owning ≥1 mobile phones	5 points ∗ % of households owning ≥1 mobile phones	0–5
Education	Presence of secondary school	2 points ∗ any secondary school in ward	0 or 2
	Percent of women with any secondary school education	8 points ∗ % of women with any secondary school education	0–8
Diversity	Variance in women’s education (y)	Decile 1	1
		Decile 2	2
		Decile 3	3
		Decile 4	4
		Decile 5	5
		Decile 6	6
		Decile 7	7
		Decile 8	8
		Decile 9	9
		Decile 10	10
Health services	Presence of pharmacy or dispensary	Facility located in ward	10
		Facility located in district	5
		No facility in district	0

1 Lower % of households with ≥1 adult working in agriculture results in a higher domain score.

### Urbanicity scale scoring and validation

The scale was assessed using equal and unequal weighting schema across the 8 domains; for the unequal schema, the markets domain was weighted less than the other domains, as it included only 2 variables that were thought to hold less theoretical significance toward urbanicity than the other domains. Pearson’s correlation coefficient was used to measure the degree of overlap between the equal and unequal weighting schema. An equally weighted scoring schema (all domains weighted equally) was chosen for the final scale, as the unequal and equally weighted schema were nearly perfectly correlated (*r* = 0.99, *P* < 0.05), and as there is a precedent for using equal weighted domains in other urbanicity scales. The final scoring schema is outlined in Table 1.

For validation purposes, factor analysis was used to determine unidimensionality, or whether the 8 domains of the urbanicity scale measured 1 latent construct (urbanicity). Internal consistency was assessed by calculating item-test correlations for each domain. Face validity of ward’s urbanicity scores was assessed with informal conversations with original PoSHAN data collectors virtually and in Nepal, some of whom continued to follow and collect data in the same sampled communities over the following 4–5 y. These original data collectors provided consensus on the zones distribution of scores and the relative ranks of wards after considering the domains of the urbanicity scale. Construct validity was assessed by comparing the novel urbanicity scale against a housing quality index (also developed from 2013 PoSHAN data), a measure known to correlate positively with urbanicity [35]. This housing quality index was calculated as the average of 5 equally weighted variables also from PoSHAN 2013 surveys: improved house material (floor, wall, and roof), water source (indoor tap), and cooking energy source (gas compared with others). Housing quality variables were chosen based on previously established housing indices [37] and the available data in the PoSHAN 2013 survey. Linear regression modeling assessed the correlation between the urbanicity scale and the housing index.

### Statistical approach

All analyses were conducted using STATA/IC 16.1. Exploratory analyses were employed to assess the distributions of community urbanicity, food insecurity, and agricultural occupation variables. Households with missing responses to food insecurity or agricultural occupation variables (<1% of observations) were excluded from the analysis. ∼10% of observations for the socioeconomic status variable were missing, so a missing indicator dummy variable was generated and included in the analyses.

Multilevel mixed effects logistic regression analysis was used to model household level food insecurity (outcome) as a function of community urbanicity (exposure), accounting for potential outcome clustering within communities. A threshold (alpha-level) of ≤ 0.05 was used for assessing statistical significance. All models included the continuous urbanicity scale variable. Potential confounders were selected based on a review of the literature and were included in the multivariate model if they remained statistically significant in forward stepwise covariate selection. Final adjusted models included agroecological zone, household’s main water source, number of household members, whether households produced any crops during the dry or rainy season, head of household’s age, sex, marital status, religion, ethnicity/caste, agricultural occupation, and school years completed. An interaction term between community urbanicity and agricultural occupation was added to the unadjusted model, to test for the difference in odds of food insecurity between groups (having an agricultural occupation compared with other occupation).

## Results

### Urbanicity across agroecological zones

**FIGURE 1:**
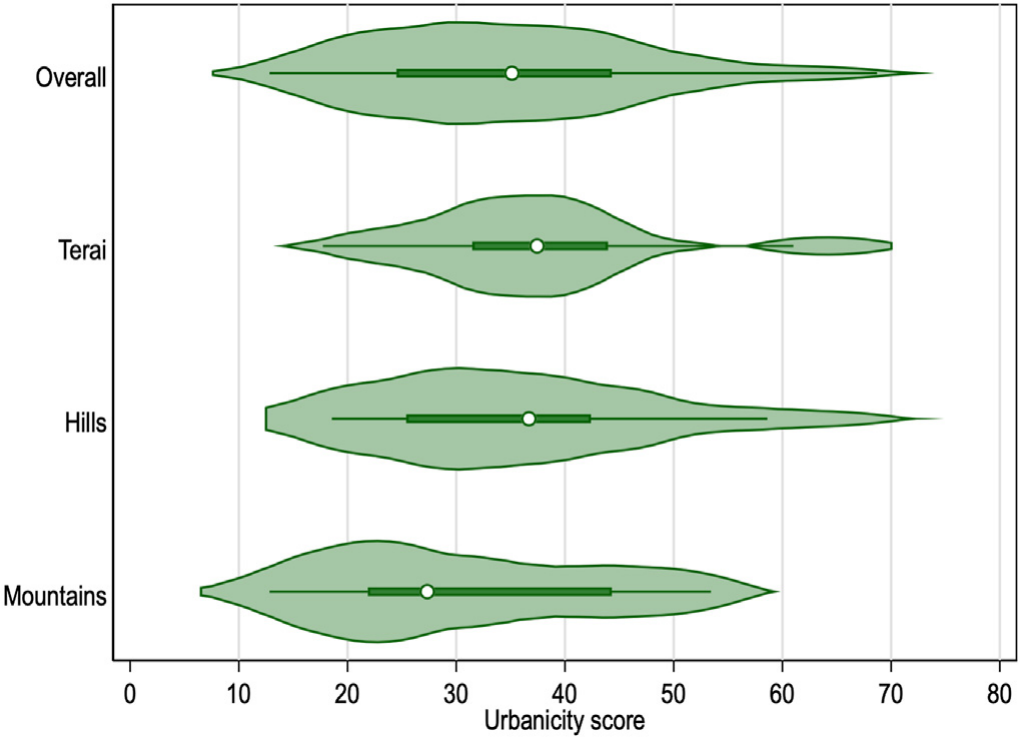
Violin plot of urbanicity scores, overall and by agroecological zone. Each horizontal plot shows the distribution of urbanicity scores for communities (wards) overall at the top, and then stratified by the 3 agroecological zones below. Violin plots combine basic summary statistics of a box plot with visual information of a local density estimator, to reveal the distributional structure in a variable—in this case, urbanicity scores ranging from 0 to 80 for each agroecological zone, and nationally (overall). For each of the 4 plots, the white dot indicates the median community urbanicity score, the thick green horizontal line indicates the interquartile range, and the thin green horizontal line indicates the upper- and lower-adjusted values. Overlaid for each of the 4 plots is a density (green space), estimated by kdensity.

**FIGURE 2:**
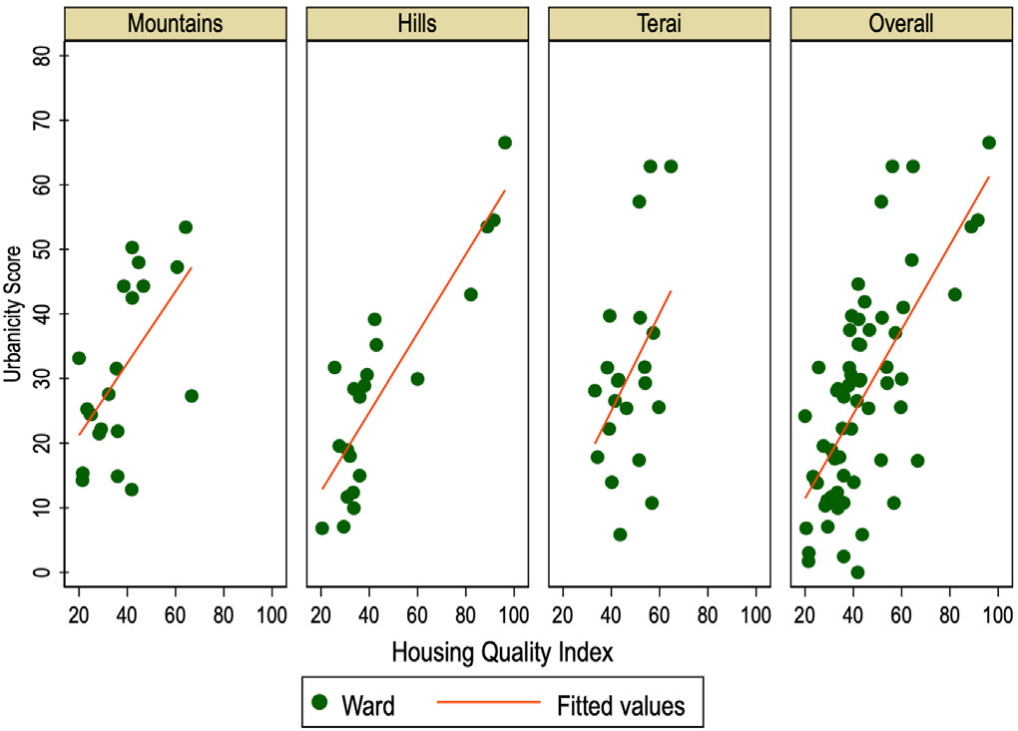
Scatterplot, housing quality index predicting urbanicity scale. This scatterplot shows communities (green dots) plotted by their urbanicity score (ranging 0–80) and housing quality index score (ranging 0–100), for each agroecological zone and overall. This is a visual illustration of an overlaid linear prediction plot, indicated with the red line, where housing quality was found to positively predict urbanicity in each agroecological zone, and overall (nationally).

Urbanicity scores for each community ranged between 12.82 and 68.70, out of a possible 80 points, across the 63 communities. Summary statistics are shown at the national level and by agroecological zone for each variable included in the scale in Supplemental Table 2. The distribution of scores at the national level and by agroecological zone is depicted in Figure 1 and described by domain in Supplemental Table 3. Using mixed effects modeling, ICC showed ∼59% of score variance was explained by differences in districts; in other words, ∼41% of the variance in scores was explained by differences in communities. One-way ANOVA showed nonsignificant difference (*P* = 0.16) in mean urbanicity scores between the 3 agroecological zones. Item-test correlations for each domain (Supplemental Table 4) ranged from 0.48 (population density) to 0.82 (Communication), showing sound internal consistency. Linear regression showed a positive and statistically significant correlation with the housing quality index predicting the novel urbanicity scale (0.55, *P* ≤ 0.001, *R*^2^ = 0.42, Figure 2), strengthening construct validity. Figure 2 also illustrates the slight differences in this correlation by agroecological zone, with the hill zone comprised of communities scoring high in both housing quality and urbanicity that are not found elsewhere.

### Relationship between urbanicity and food insecurity

**FIGURE 3:**
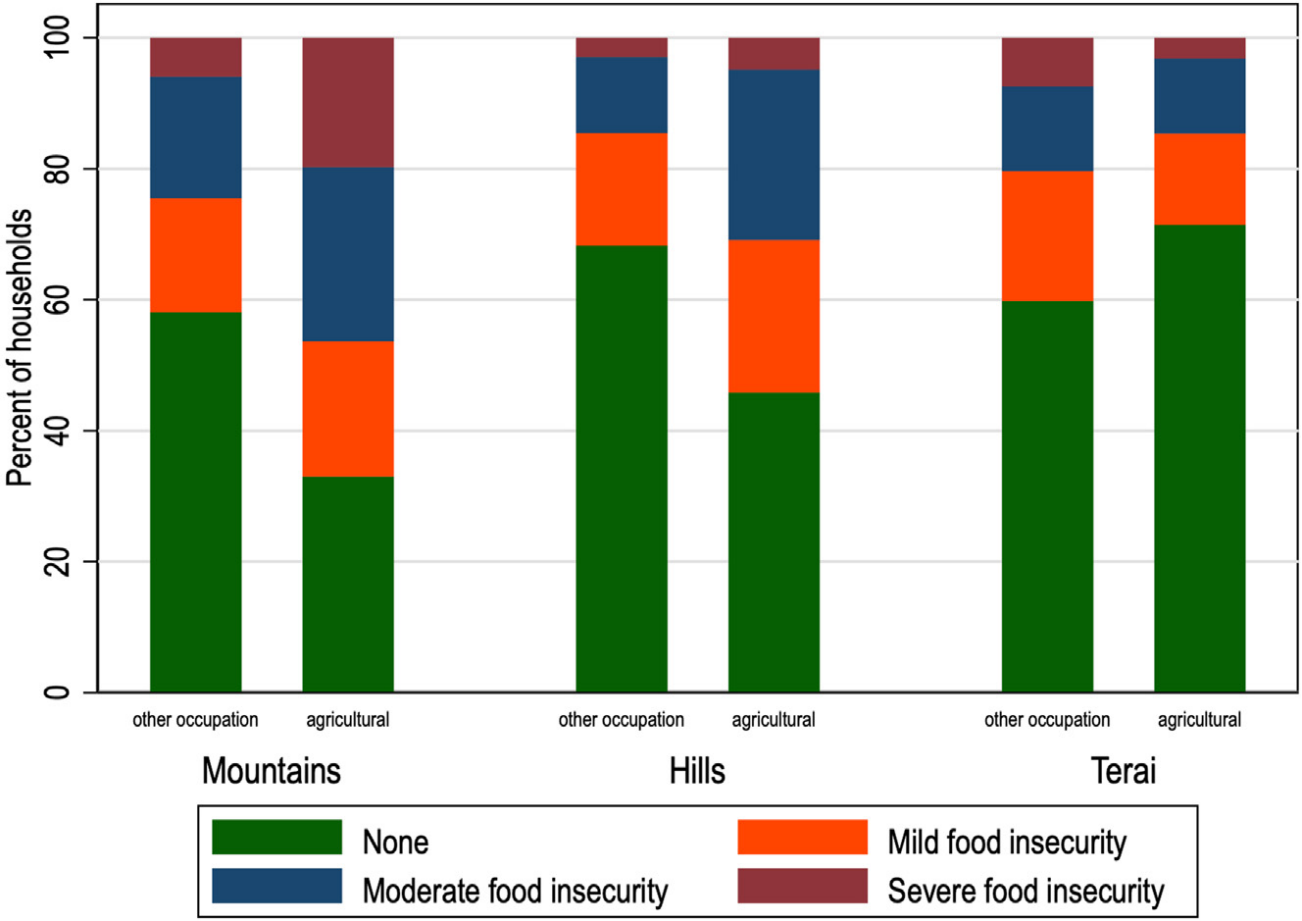
Proportion of each food security category by agroecological zone and occupation. The mountain, hill, and Terai agroecological zones are listed along the X-axis, with each zone stratified by households with heads of household working in agriculture compared with all other occupations of households. Each bar represents 100% of the sample in that subgroup. Each bar also shows the composition of that total proportion of households that are mildly, moderately, or severely food insecure, or food secure (“none”).

Of the 4284 households included in the analytical sample, over a quarter (26.4%) were headed by women. A total of 40.1% of all households were categorized as mildly, moderately, or severely food insecure, with the mountain agroecological zone showing the highest prevalence of any food insecurity (48.8%), compared with the hill and *Tarai*. Overall, almost 43% of agricultural households were food insecure, compared with 40% of all households. Figure 3 shows that a majority of agricultural households in the mountain (67%) and hill (54%) zones were categorized as food insecure (mild, moderate, or severe); the *Tarai* was the only zone where agricultural households faced a lower prevalence of food insecurity compared with other occupations. Areas with higher urbanicity had a lower proportion of agricultural households, but it remained 1 of the most highly reported professions (Supplemental Figure 3). Other summary statistics, nationally and by agroecological zone, are shown in Table 2.

**TABLE 2:** Summary statistics of analytical variables

Variables of interest		Mountains (*N* = 793) *n* (%)	Hills (*N* = 1126) *n* (%)	*Tarai* (*N* = 2365) *n* (%)	Overall (*N* = 4284) *n* (%)
Head of household’s sex	Male	625 (78.8)	672 (59.7)	1858 (78.6)	3155 (73.6)
	Female	168 (21.2)	454 (40.3)	507 (21.4)	1129 (26.4)
Head of household’s caste/ethnicity group	Brahmin/Chettri^1^	434 (54.7)	403 (35.8)	222 (9.4)	1059 (24.7)
	Other Tarai Caste	1 (0.1)	2 (0.2)	1253 (53.0)	1256 (29.3)
	Janajati	180 (22.7)	387 (34.4)	335 (14.2)	902 (21.1)
	Newar, Dalit, Others	178 (22.4)	334 (29.7)	555 (23.5)	1067 (24.9)
Household food insecurity category (HFIAS)	Food secure	406 (51.2)	653 (58.0)	1507 (63.7)	2566 (59.9)
	Mildly food insecure	145 (18.3)	225 (20.0)	422 (17.8)	792 (18.5)
	Moderately food insecure	165 (20.8)	205 (18.2)	294 (12.4)	664 (15.5)
	Severely food insecure	77 (9.7)	43 (3.8)	142 (6.0)	262 (6.1)
Agricultural occupation of head of household	Other occupation	575 (72.5)	611 (54.3)	1572 (66.5)	2758 (64.4)
	Agricultural occupation	218 (27.5)	515 (45.7)	793 (33.5)	1526 (35.6)
Community urbanicity score, median (IQR)		44.3 (27.6–47.3)	38.0 (23.2–57.8)	39.4 (35.1–61.0)	39.4 (34.2–50.3)

1 Brahmin/Chettri reflects the caste groups at the top of caste hierarchy in Nepal [52].

Multilevel mixed effects logistic regression models showed an increase in community urbanicity was negatively associated with food insecurity (unadjusted odds ratio [OR] for a 10-unit increase: 0.67; confidence interval [CI]: 0.58, 0.78; *P* ≤ 0.05). Stratified by the 3 agroecological zones, as shown in Table 3, the unadjusted relationship remained statistically significant in the mountain zone (OR for a 10-unit increase: 0.65; CI: 0.49, 0.85; *P* ≤ 0.05) and hill zone (OR for a 10-unit increase: 0.59; CI: 0.46, 0.74; *P* < 0.05). In the *Tarai*, community urbanicity was not a significant predictor of food insecurity (OR for a 10-unit increase: 0.81; CI: 0.64, 1.02; *P* = 0.08). Supplemental Figure 4 illustrates this relationship in each of the agroecological zones, using the continuous HFIAS score; the fitted line slopes differently in each zone, with the *Tarai* slope being most horizontal, demonstrating the smaller association between community urbanicity and food insecurity in this zone.

**TABLE 3:** Results from logistic regression, urbanicity predicting food insecurity

	Unadjusted			Adjusted		
	Oddsratio^1^	95%CI	*P*value	Oddsratio	95% CI	*P* value
Overall	0.67	0.58, 0.78	0.00	0.82	0.71, 0.94	<0.001
Mountains	0.65	0.49, 0.85	0.002	0.71	0.54, 0.92	0.010
Hills	0.59	0.46, 0.74	0.000	0.80	0.64, 1.00	0.051
*Tarai*	0.81	0.64, 1.02	0.079	0.91	0.74, 1.13	0.386

Abbreviations: CI, confidence interval.

1 An odds ratio (OR) < 1.00 indicates a decrease in the odds of the event occurring. The overall unadjusted OR = 0.67, interpreted as a 33% decrease in the odds of food insecurity occurring for each 10-unit increase in urbanicity.

After adjusting for potential confounders, there was a 18% decrease in the odds of food insecurity for every 10-unit increase in community urbanicity score, holding other factors constant (OR for a 10-unit increase: 0.82; CI: 0.71, 0.94; *P* ≤ 0.05). Variance inflation factors (VIF) were used to test for collinearity of variables in the adjusted model, with a mean VIF of 3.38 which did not suggest multicollinearity concerns among independent variables. Agroecological zones did not statistically significantly differ from each other in the fully adjusted model (*P* > 0.05). Agricultural occupation was not found to statistically significantly modify the unadjusted nor the fully adjusted associations between urbanicity and household food security; the interaction term (community urbanicity and occupation group) in the overall unadjusted model did not show a significant difference between the occupation groups (OR: 0.89; 95% CI: 0.78, 1.00; *P* = 0.07). However, the estimated (unadjusted and adjusted) associations were more substantial in the mountains and hill zones as compared with the Tarai.

## Discussion

This study is the first to explore the association between a novel community urbanicity scale and food insecurity in a nationally representative sample. It showed that greater community urbanicity predicted lower odds of household food insecurity in Nepal overall; this relationship was the strongest in the mountain zone. Agricultural occupation was not a significant effect modifier of the relationship. The magnitude of the relationship between urbanicity and food security was similar across all 3 zones, and there was notable overlap in the distribution of urbanicity scores across zones despite different topography and built infrastructure. The *Tarai* zone was expected to differ from the mountain and hills in terms of its urbanicity-to-food security relationship, as its districts are rapidly increasing in population density, and because of their connectivity to road and market infrastructure [38,39] but this was not supported by the findings.

A central finding in applying the urbanicity scale was that Nepal’s agroecological zones did not significantly differ from 1 another, even though Nepal’s agroecological zones have historically been understood to vary distinctly regarding population density and infrastructure [24]. This study combined multiple domains understood to comprise urbanicity, capturing a wider distribution within each of the zones than that was previously seen using binary urban compared with rural measures.

Most literature linking greater food security to urbanicity use the dichotomous urban compared with rural measure at the country level [40], and report similar directionality as we found using the continuous scale. However, most global-level food security data, such as estimates reported by the FAO of the United Nations, lack sufficient stratified sample sizes to disaggregate by urban compared with rural areas [41]. The challenge of exploring community urbanicity’s influence on food security stems also from unstandardized measurements; most countries report “urbanness” using the binary urban compared with rural classification, but these are calculated using different variables and thresholds in each country, making cross-country comparisons challenging [1]. Some global analyses have used other methods to characterize urbanicity to report food insecurity, such as Gallup World Poll’s survey that asks respondents to self-report whether they lived in a rural area or farm, small town or village, suburb of a large city, or a large city [10]; studies using these categories show the prevalence of food insecurity is higher in more rural areas [42].

Head of household’s agricultural occupation was a component of the urbanicity scale and was also explored as a potential effect modifier of the relationship between urbanicity and food security. Agricultural households in this study faced lower odds of food insecurity as urbanicity increased, though this relationship did not hold when controlling for other factors. It is important to note that an increase in urbanicity is by definition linked to a decrease in agricultural occupation (the novel scale includes a variable for agricultural occupation), and so those more urban households that are involved in agriculture may do so by choice, reflecting a greater well-being and household food situation [17]. Globally, interlinkages between food security and agricultural livelihoods are recognized as bidirectional [17,43,44], and these exchanges are observable within the agroecological zones of Nepal.

This study suggests that being involved in agricultural work means different things depending on where 1 lives in Nepal—agricultural occupation provides a slight protection against food insecurity in the *Tarai* zone, and a slight risk in the mountain and hill zones, which likely reflects greater access to agriculturally productive land in the Tarai. Yet, measuring agricultural occupation is particularly complex in this setting; Nepalese women are more likely than men to work in agriculture, but they are less likely to receive payment for their work [14]. In neighboring India, women’s agricultural tasks were often not remunerative, or considered “work,” and were linked in part to negative status implications [45,46]. One qualitative study in Nepal’s hill zone found that off-farm work opportunities improved food purchasing for some, whereas patterns of unequal and absentee land ownership, women’s overwork, and women’s trends away from agriculture erode local, small-scale agriculture [47]. For these more agrarian-reliant areas, greater recognition of livelihood diversity that includes agricultural work is needed, as is policy reform where rapid structural changes in land use and food production, distribution, and market access are occurring [48]. These cultural norms and informal work structures can inform and improve employment and occupation surveys, to better measure and understand (women’s) agricultural occupation [44,49].

The variability of the urbanicity score components (as opposed to total scores) illustrates how the 8 domains drive the urbanicity score in different zones. This is an important insight compared with what is observable using the traditional urban–rural binary, which lacks the many factors understood to comprise urbanicity, such as communication, diversity, and infrastructure [22]. The current scale indicated a slight inverse correlation between population density and Diversity domains (in Supplemental Figure 1). This was interpreted as a sign that the right “noise” is being captured, or nuance is being measured across these domains that drive urbanicity scores at the national level; zones vastly differed by population density and educational variance, and this slight inverse correlation is interpreted as accurately reflecting less correlation in those zones, but overall remaining an integral part of the urbanicity measure. Similarly, different domains “drove” the urbanicity score by zone; for example, population density and economic activity drove scores higher in the *Tarai*, built environment in the hills, and diversity in the mountains.

There were some limitations to this analysis. One limitation of the dataset was the sampling exclusion of households without children ≤ 5 y of age or a recently married couple, therefore limiting the generalization to households within this criteria, or ∼64% of the 2011 population aged 10 y and older [24]. For the purposes of this research question, 1 limitation of the 2011 Nepal Census was that population density was available only at the district level, resulting in each of the 3 communities (wards) located within the same district being assigned the same population density value (though the actual density is expected to vary by ward). Another limitation of this analysis was the characterization of agricultural occupations in the household, which was limited to the head of household and did not capture agricultural livelihood engagement of other household members. As such, it may miss diversity in agricultural livelihoods at the household level.

Another limitation of the study design, for the purposes of this research, was that the PoSHAN study sample primarily consisted of rural VDCs, a result of the stratified systematic random sampling approach employed to ensure an agro-ecologically and nationally representative sample. If more urban places had been randomly selected, this may have added nuance to the food security connection, which may have differed from similar, but slightly less urban places. Still, the urbanicity gradient we offer here is understood to represent the relationship with food security at different levels of urbanness; we would expect that a more recent community distribution will see more urban localities. Another limitation was the 10-y time gap between the first year of PoSHAN data collection and this study. Though Nepal has continued to rapidly urbanize because data collection began in 2013, this study adds to the literature by showing a wider distribution of urbanicity than that had previously been documented for communities at that time, using existing and high-quality data. Future studies can build from this urbanicity scale and its relationship to household food security by collecting more targeted data for improved scale performance and finer representation of communities in real time.

Still, many strengths allowed for a novel investigation, notably the PoSHAN study sampling strategy, designed to represent the diversity observed across the mountains, hills, and Tarai while also providing a nationally representative sample. Furthermore, all eligible households were enumerated within sampled wards, which enabled community-level investigation of factors of interest and is a unique aspect of in-depth, comprehensive surveys such as those included in the PoSHAN study.

This article demonstrates that a novel urbanicity scale can be created and validated using common, publicly available data in a low-income context such as Nepal, and that applying such a scale can provide useful insights and inform policy. Systematic reviews call for a greater use of urbanicity scales, especially in low- and middle-income countries [22,23], to more accurately inform policies and governance structures, thereby influencing an area’s resources and services able to promote health [4,50]. The importance of using a refined urbanicity measurement is furthered by rapid urbanization rates seen in countries like Nepal, often overlapping with populations facing chronic food insecurity. This study supports the view of urbanization not as an autonomous process onto food systems and agriculture, but rather as a process dependent on and (re)shaped by rural developments and resource use [51], and further implicates nuanced urbanicity measurement as crucial for measuring and mitigating food insecurity.

## Acknowledgments

We are very grateful for the participation of PoSHAN study respondents. We also acknowledge the significant contributions of the New ERA and Nepali Technical Assistance Group data collection teams, and the Nutrition Innovation Lab-Nepal/JHU research team. We also acknowledge the support from the Government of Nepal for the parent study and the leadership and partnership of Tufts University.

### Author contributions

The authors’ responsibilities were as follows – EG, ATL, JM: designed the research question and analysis; KW, SM: designed the PoSHAN study; SM: oversaw data collection; SN: conducted data curation and collection; EG: analyzed the data and wrote the initial draft of the manuscript; ATL, JF, JM, SM, YM: critically reviewed the manuscript; EG: had primary responsibility for the final content; and all authors: read and approved the final manuscript.

### Conflict of interest

The authors report no conflicts of interest.

### Funding

This analysis was supported through doctoral awards to EG through Johns Hopkins University, including the Proctor & Gamble Fellowship. Support for this paper was provided by the Feed the Future Food Systems for Nutrition Innovation Lab at Tufts University, cooperative agreement number 7200AA21LE0001 and the Nutrition Innovation Lab, cooperative agreement number AID-OAA-L-10-00006, both funded by the United States Agency for International Development (USAID). The opinions expressed herein are solely those of the authors.

### Data availability

Data described in the manuscript, code book, and analytic code will be made available upon request pending application and approval.

## Figures and Tables

**Figure:**
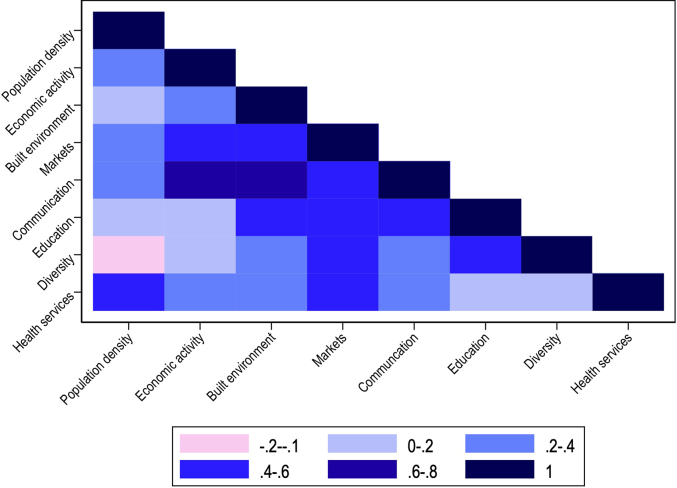


**Figure:**
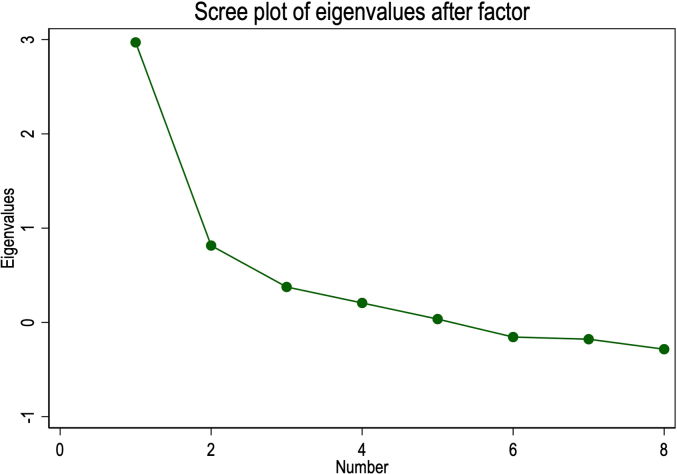


**Figure:**
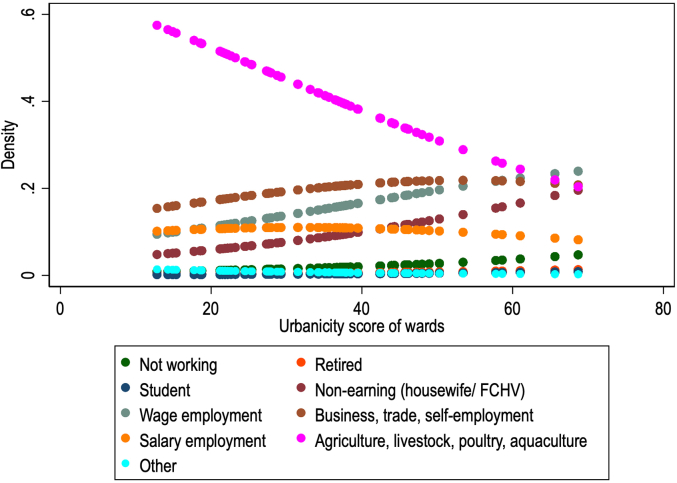


**Figure:**