# Supplementary material for: Measuring Community Urbanicity and Its Influence on Household Food Security Across Nepal’s Agroecological Zones
Source: Curr Dev Nutr. 2024 May 14;8(6):103773. doi: 10.1016/j.cdnut.2024.103773 (PMC11214172; doi:10.1016/j.cdnut.2024.103773)
Supplement: Multimedia component 1 [file mmc1.docx]

**Supplementary Tables**

**Supplementary Table 1** Full list of original variables considered for urbanicity scale, by scale iteration

| **Domain** | **Variables in every scale iteration** | **Variables that interchange across scale iterations** | **Scale iterations** | | | | | |
| --- | --- | --- | --- | --- | --- | --- | --- | --- |
|  |  |  | **11** | **22** | **33** | **44** | **55** | **66** |
| **Population density** | *1. Mean population density (individuals per square km) |  |  |  |  |  |  |  |
| **Economic activity** | 2. | 2a. Total adults with primary agricultural occupation |  |  |  |  |  |  |
|  |  | 2b. Head of HH with primary agricultural occupation |  |  |  |  |  |  |
|  |  | *2c. HH with >1 adults with primary agricultural occupation |  |  |  |  |  |  |
| **Built environment** | *3. Paved road(s) |  |  |  |  |  |  |  |
|  | *4. Bus stop(s) |  |  |  |  |  |  |  |
|  | *5. % of HH with flush toilet |  |  |  |  |  |  |  |
|  | *6. % of HH with electricity |  |  |  |  |  |  |  |
| **Markets** | *7. Permanent bazaar(s) |  |  |  |  |  |  |  |
|  | *8. Haat bazaar market(s) |  |  |  |  |  |  |  |
| **Communication** | *9. % of HH owning >1 TVs |  |  |  |  |  |  |  |
|  | 10. | *10a. % of HH owning >1 mobile phone |  |  |  |  |  |  |
|  |  | 10b. % of women owning >1 mobile phone |  |  |  |  |  |  |
|  | 11. % of HH owning >1 radio |  |  |  |  |  |  |  |
| **Education** | 12. Primary school(s) |  |  |  |  |  |  |  |
|  | *13. Secondary school(s) |  |  |  |  |  |  |  |
|  | *14. % of women with any secondary education |  |  |  |  |  |  |  |
| **Diversity** | *15. Variance in women’s education |  |  |  |  |  |  |  |
| **Health services** | 16. Sub-Health Post(s) |  |  |  |  |  |  |  |
|  | 17. Health Post(s) |  |  |  |  |  |  |  |
|  | 18. Private hospital or clinic |  |  |  |  |  |  |  |
|  | *19. Pharmacy or dispensary |  |  |  |  |  |  |  |
|  | 20. Number of nurses and health workers |  |  |  |  |  |  |  |

* Included in the final 14-variable scale

**Supplementary Table 2** Summary of urbanicity scale indicators by domain and agroecological zone. Includes all variables used to construct the urbanicity scale.

| **Domain** | **Indicator** | **Overall** | **Mountains** | **Hills** | ***Tarai*** |
| --- | --- | --- | --- | --- | --- |
|  | *# of wards (communities)* | *63* | *21* | *21* | *21* |
| **Population density** | Population density at district level (Mean [SD]) | 420.77 | 46.3 [30.4] | 740.9 [1537.6] | 475.1 [154.9] |
| **Economic activity** | Percent of households with >1 more adults reporting agricultural occupation (Mean [SD]) | 55.22% | 57.03% [33.29] | 63.46% [30.65] | 45.17% [22.15] |
| **Built environment** | Paved roads (n) | 21 | 3 | 5 | 13 |
|  | Bus stops (n) | 29 | 5 | 16 | 8 |
|  | Percent of households with flush toilet (Mean [SD]) | 29.98% [33.6] | 17.19% [19.13] | 54.83% [42.61] | 17.92% [18.56] |
|  | Percent of households with electricity (Mean [SD]) | 82.86% [22.56] | 93.24% [13.64] | 76.31% [30.47] | 79.02% [17.02] |
| **Markets** | Permanent bazaars (n) | 32 | 10 | 17 | 5 |
|  | Haat bazaars (n) | 8 | 1 | 0 | 7 |
| **Communication** | Percent of households owning 1 or more TVs (Mean [SD]) | 34.68% [25.78] | 31.42% [24.81] | 32.18% [30.98] | 40.45% [20.79] |
|  | Percent of households owning 1 or more mobile phones (Mean [SD]) | 85.49% [13.01] | 81.04% [15.96] | 89.69% [9.21] | 85.74% [12.06] |
| **Education** | Secondary schools  N (% of wards with a school) | 19 | 2 (9.5%) | 8 (38.1%) | 9 (42.86%) |
|  | Percent of women with any secondary school education | 35.31% [23.22] | 30.18% [18.29] | 48.37% [24.13] | 27.26% [21.93] |
| **Diversity** | Variance in women’s education (Mean [SD]) | 15.93 [7.48] | 19.41 [9.49] | 15.12 [4.9] | 13.26 [6.21] |
| **Health services** | Pharmacy or dispensary | 55 | 10 | 14 | 31 |

**Supplementary Table 3** Summary of scale domain scores and total scale score, overall and by agroecological zone

|  | | **Overall** | **Mountains** | **Hills** | ***Tarai*** |
| --- | --- | --- | --- | --- | --- |
| **Domain** | | *Mean (SD)* | *Mean (SD)* | *Mean (SD)* | *Mean (SD)* |
| Population density | | 4.2 (3.0) | 1.4 (0.8) | 4.0 (2.6) | 7.3 (1.6) |
| Economic activity | | 4.5 (3.0) | 4.3 (3.3) | 3.7 (3.1) | 5.5 (2.2) |
| Built environment | | 4.8 (2.1) | 4.6 (1.4) | 5.5 (2.9) | 4.2 (1.5) |
| Markets | | 2.0 (3.1) | 1.9 (3.0) | 2.1 (2.5) | 1.9 (3.7) |
| Communication | | 6.0 (1.7) | 5.6 (1.8) | 6.1 (1.9) | 6.3 (1.5) |
| Education | | 3.4 (2.1) | 2.6 (1.8) | 4.6 (2.1) | 3.1 (2.0) |
| Diversity | | 5.5 (2.9) | 6.8 (3.3) | 5.3 (2.1) | 4.5 (2.8) |
| Health services | | 4.8 (3.4) | 3.6 (3.6) | 5.0 (2.7) | 6.0 (3.4) |
| **Scale total** | **Mean (SD)** | **35.3 (13.5)** | **30.8 (13.1)** | **36.3 (13.8)** | **38.6 (13.0)** |
|  | Min - Max | 12.8 - 68.7 | 12.8 – 53.4 | 18.6 – 68.7 | 17.7 – 65.6 |

Supplementary Table 4 Item-test correlation for urbanicity domains

| **Domain** | **Item-test correlation** |
| --- | --- |
| Population Density | 0.4819 |
| Economic activity | 0.6516 |
| Built environment | 0.7103 |
| Markets | 0.7437 |
| Communication | 0.8232 |
| Education | 0.6653 |
| Diversity | 0.5099 |
| Health services | 0.5838 |

**Supplementary Figure 1** Correlation matrix of scale domains. The eight domains that comprise the novel urbanicity scale are shown on both X and Y axes, as this matrix is to illustrate the correlation that each domain has with all other domains within the scale. The darkest blue diagonal boxes represent a perfect correlation – each domain’s correlation with itself. The other colored boxes range form -0.2 – 0.8. While most domains are positively and highly correlated, one relationship is slightly inversely correlated: diversity and population density domains. This is attributed to the study sample of the Tarai being larger than the other agroecological zones, and knowing that in the Tarai, diversity scores were lower than the other zones. These Tarai communities would be considered urban if single indicator measurement (population density) was used. However, considering more indicators (the eight domains comprising our urbanicity scale including diversity) the Tarai communities are understood to have rural features as well, placing them lower on the urbanicity scale than other places with mostly urban features. This inverse relationship with a few domains is expected and even hoped for when assessing the scale’s performance, as the intention of the scale is to show nuance in communities across these domains empirically representing urbanicity.

**Supplementary Figure 2** Scree plot of eigenvalues. The factor analysis to test our urbanicity scale’s eight domains for unidimensionality resulted in the first factor having a high eigen value (> 1) with all subsequent factors below 1, after PCA. This suggests that the scale’s domains measure a latent unidimensional construct of urbanicity.

**Supplementary Figure 3** Households reporting occupations across the urbanicity gradient. The Y axis shows the density of households (.2 = 20% of all households at that urbanicity gradient). The X axis shows the urbanicity gradient, ranging from 0 – 80. The nine categories of occupation for head of household are plotted, with most remaining consistent across the urbanicity spectrum. Of course, the only agricultural occupation was inversely correlated with a rise in urbanicity – as expected from its inclusion in the urbanicity scale itself. However, agriculture remains one of the most densely reported occupations in the most urban communities. This suggests that the use of scales (multiple domains or indicators) versus the single indicator of agricultural occupation as is used in many countries including Nepal, can better describe a community’s urbanness and represent it along the urbanicity gradient.

**Supplementary Figure 4** HFIAS score across urbanicity gradient, by agroecological zones. Four panels show the three agroecological zones and a total (national) scatter plot. The Y axis shows household food security, measured using the Household Food Insecurity Access Scale (HFIAS) continuous score, ranging from 0-27. The X axis for each panel shows the urbanicity score, ranging 0 -80. This figure illustrates the unadjusted relationship of urbanicity predicting household food security, which was found to be significant in the mountain and hill zones, but not in the Tarai agroecological zone. While this finding does not adjust for confounding factors, it may suggest a smaller and different association between community urbanicity and household food security in the Tarai, compared to the other agroecological zones.
